# Supplementary material for: Association of Renin-Angiotensin System Blockers with Survival in Patients on Maintenance Hemodialysis
Source: J Clin Med. 2023 May 5;12(9):3301. doi: 10.3390/jcm12093301 (PMC10179028; doi:10.3390/jcm12093301)
Supplement: Supplementary file 1 [file jcm-12-03301-s001.zip › jcm-2295735-Supplementary material.pdf]

Supplementary Material

Table S1. Medication types and Health Insurance Review and Assessment Service codes

Table S2. ICD-10 codes of Charlson Comorbidity Index

Table S3. Cox regression analyses for patient survival according the use of RAS blockers and β-blockers in Groups 2 and 3

Table S4. Patients' clinical characteristics after weighting

Table S5. Cox regression analyses for patient survival using weighted data

Figure S1. Study flow diagram

Figure S2. Kaplan–Meier curves of patient survival according the use of RAS blockers and β-blocker in Groups 2 and 3

Figure S3. Absolute standardized difference plots for estimating propensity scores to generate weights

Figure S4. Kaplan–Meier curves of patient survival according to groups using weighted data

Table S1. Medication types and Health Insurance Review and Assessment Service codes.

Medications	Codes
**Acepril**	104201ATB, 104202ATB
**Amlodipine**	495901ATB, 459802ACH, 483201ATB, 486501ATB, 107601ATB, 107601ATD, 459801ACH, 459801ATB, 459901ATB, 464601ATB, 470801ATB, 476201ATB, 479701ATB, 483202ATB, 486502ATB, 107602ATB, 107602ATD, 470802ATB
**Amlodipine+Atorvastatin**	614500ATB, 472300ATB472400ATB472500ATB518900ATB
**Amlodipine+Losartan+Chlorthalidone**	662800ATB, 662900ATB, 663000ATB
**Amlodipine+Losartan+Rosuvastatin**	663900ATB, 664000ATB, 664100ATB, 664200ATB, 664300ATB, 664400ATB
**Amlodipine+Olmesartan+Rosuvastatin**	677300ATB, 677400ATB, 677500ATB, 677600ATB
**Amlodipine+Rosuvastatin**	673900ATB, 674000ATB, 674100ATB
**Amlodipine+Rosuvastatin+Telmisartan**	671200ATB, 671300ATB, 671400ATB, 671500ATB, 677000ATB, 677100ATB, 671600ATB, 671700ATB
**Amlodipine+Telmisartan+Hydrochlorothiazide**	663500ATB, 663600ATB, 663700ATB, 663800ATB
**Amosulalol**	107901ATB, 107902ATB
**Arotinolol**	110202ATB, 110201ATB
**Atenolol**	483102ATB, 111402ATB, 483101ATB, 111403ATB, 111401ATB
**Atenolol+Chlorthalidone**	262100ATB, 460200ATB
**Azilsartan**	662401ATB, 662403ATB, 662402ATB
**Azilsartan+Chlorthalidone**	673500ATB, 673600ATB
**Barnidipine**	114003ACH, 114001ACH, 114002ACH
**Benidipine**	115101ATB, 115102ATB, 115104ATB, 115103ATB
**Betaxolol**	116801ATB, 116803ATB
**Bevantolol**	117002ATB, 117001ATB
**Bisoprolol**	117904ATB, 117903ATB, 117902ATB, 117901ATB
**Bisoprolol+Hydrochlorothiazide**	469800ATB, 470000ATB, 469900ATB
**Candesartan**	122601ATB, 122602ATB, 122603ATB
**Candesartan+Amlodipine**	652900ATB, 653000ATB, 653100ATB
**Candesartan+Hydrochlorothiazide**	423700ATB
**Candesartan+Rosuvastatin**	661800ATB, 661900ATB, 673700ATB, 662000ATB, 662100ATB
**Captopril**	122901ATB, 122902ATB, 122903ATB
**Captopril+Hydrochlorothiazide**	262200ATB, 262300ATB
**Carteolol**	124801ATB
**Carvedilol**	125005ATB, 125003ATB, 662201ATB, 125008ACR, 125001ATB, 662202ATB, 125007ACR, 125002ATB, 125006ACR, 125004ACR

**Celiprolol**	129101ATB
**Cilazapril**	133001ATB, 133002ATB, 133003ATB
**Cilnidipine**	133102ATB, 133101ATB
**Clonidine**	136505ATR
**Diltiazem**	145706ATB, 145707ACR, 145707ATR, 145703ACR, 145706ATR, 145707ATB
**Doxazocin**	149101ATB, 149102ATB, 149104ATR, 149103ATB
**Efonidipine**	441202ATB, 441201ATB
**Enalapril**	151603ATB, 151601ATB
**Enalapril+Hydrochlorothiazide**	453700ATB, 440300ATB
**Eprosartan**	429201ATB
**Eprosartan+Hydrochlorothiazide**	460500ATB
**Felodipine**	157503ATR, 157501ATR
**Felodipine+Metoprolol**	262400ATR
**Fimasartan**	515203ATB, 515201ATB, 515202ATB
**Fimasartan+Amlodipine**	651900ATB, 652000ATB, 652700ATB, 652100ATB
**Fimasartan+Hydrochlorothiazide**	522000ATB, 526800ATB
**Fimasartan+Rosuvastatin**	655000ATB, 654900ATB, 654800ATB, 654700ATB, 654600ATB
**Fosinopril**	163501ATB, 163502ATB
**Hydralazine**	170701ATB
**Imidapril**	173402ATB, 173401ATB
**Irbesartan**	177301ATB, 177303ATB
**Irbesartan+Atorvastatin**	524000ATB, 524100ATB, 527100ATB, 527000ATB
**Irbesartan+Hydrochlorothiazide**	385700ATB, 385800ATB, 553800ATB
**Lacidipine**	180301ATB, 180302ATB, 180303ATB
**Lercanidipine**	182001ATB, 182002ATB
**Lisinopril**	184501ATB
**Lisinopril+Hydrochlorothiazide**	499200ATB, 499300ATB
**Losartan**	185701ATB, 185702ATB
**Losartan+Amlodipine**	503000ATB, 637400ATB, 513900ATB, 637500ATB, 502700ATB, 637600ATB
**Losartan+Hydrochlorothiazide**	262500ATB, 486900ATB, 378900ATB
**Manidipine**	188001ATB, 188002ATB
**Metoprolol**	194003ATR, 193802ATB, 262400ATR
**Metoprolol+Hydrochlorothiazide**	262600ATB
**Metoprolol+felodipine**	262400ATR
**Minoxidil**	196102ATB

**Nadolol**	198301ATB
**Nicardipine**	201003ACR, 201002ATB
**Nifedipine**	201407ACS, 201405ATR, 528201ATR, 201409ATR, 528202ATR, 201401ACS, 201401ATB, 201408ATR
**Nimodipine**	201901ATB, 356202ATR, 356203ATR, 356201ATB, 356202ATB
**Nisoldipine**	356202ATR
**Olmesartan**	468502ATB, 468501ATB, 468503ATB, 520902ATB, 520901ATB
**Olmesartan+Amlodipine**	547800ATB, 632800ATB, 500500ATB, 547700ATB, 629500ATB, 631300ATB, 500600ATB, 547900ATB, 632900ATB, 547600ATB, 548000ATB, 582200ATB, 629600ATB, 633000ATB, 547500ATB, 582400ATB, 629400ATB
**Olmesartan+Amlodipine+Hydrochlorothiazide**	519800ATB, 519700ATB, 520100ATB, 520000ATB, 519900ATB
**Olmesartan+Hydrochlorothiazide**	513600ATB, 489100ATB
**Olmesartan+Rosuvastatin**	644200ATB, 644100ATB, 526900ATB, 526300ATB, 526400ATB, 653200ATB, 526500ATB
**Perindopril**	211301ATB, 501601ATB, 211302ATB, 501602ATB
**Perindopril+Indapamide**	556200ATB
**Propranolol**	219901ATB, 219904ATB, 219906ACR, 219905ACR
**Quinapril**	221901ATB
**Ramipril**	222401ATB, 222402ATB, 222404ATB
**Ramipril+Felodipine**	447100ATB, 447200ATB
**Ramipril+Hydrochlorothiazide**	448600ATB, 448700ATB
**Telmisartan**	378801ATB, 378802ATB, 378803ATB
**Telmisartan+Amlodipine**	521200ATB, 511600ATB, 521300ATB, 511700ATB, 521400ATB, 511500ATB, 644800ATB, 623100ATB
**Telmisartan+Hydrochlorothiazide**	443200ATB, 443300ATB, 502600ATB
**Telmisartan+Rosuvastatin**	631600ATB, 629900ATB, 630000ATB, 631700ATB, 630100ATB, 630200ATB
**Temocapril**	235002ATB
**Terazosin**	235501ATB, 235502ATB, 235503ATB, 616501ATB
**Valsartan**	247103ATB, 247101ATB, 247102ATB, 247104ATB
**Valsartan+Amlodipine**	522600ATB, 492900ATB, 522900ATB, 523200ATB, 522700ATB, 492800ATB, 522800ATB, 523000ATB, 523300ATB, 495800ATB, 523100ATB, 523400ATB
**Valsartan+Hydrochlorothiazide**	356400ATB, 442600ATB
**Valsartan+Lercanidipine**	522200ATB, 522300ATB, 522400ATB
**Valsartan+Pitavastatin**	635000ATB, 635200ATB, 634900ATB, 635100ATB
**Valsartan+Rosuvastatin**	629700ATB, 525000ATB, 525200ATB, 629800ATB, 525100ATB, 525300ATB
**Valsartan+Sacubitril**	651401ATB, 651402ATB, 651403ATB
**Verapamil**	247606ATB, 247607ATB, 247603ATR, 247605ATR, 247601ACR
**Zofenopril**	510401ATB, 510402ATB, 510403ATB

**Atorvastatin**	111501ATB, 111502ATB, 111503ATB, 111504ATB, 502201ATB, 502202ATB, 502203ATB, 502204ATB
**Atorvastatin+Amlodipine**	472300ATB, 472400ATB
**Atorvastatin+Ezetimibe**	633800ATB, 633900ATB, 634800ATB
**Fluvastatin**	162401ACH, 162402ACH, 162403ATR
**Lovastatin**	185801ATB
**Pitavastatin**	470901ATB, 470902ATB, 470903ATB
**Pitavastatin+Fenofibrate**	679300ACH
**Pravastatin**	216601ATB, 216602ATB, 216603ATB, 216604ATB
**Rosuvastatin**	454001ATB, 454002ATD, 454002ATB, 454003ATB, 454003ATD, 454005ATB
**Rosuvastatin+Ezetimibe**	640700ATB, 640800ATB, 640900ATB
**Rosuvastatin+Ezetimibe+Telmisartan**	671400ATB, 671500ATB, 671700ATB
**Simvastatin**	227801ATB, 227802ATB, 227803ATB, 227805ATB, 227806ATB
**Aspirin**	110701ATB, 110702ATB, 110801ATB, 110802ATB, 111001ACE, 111001ATB, 111001ATE, 111002ATE, 111003ACE, 111003ATE
**Clopidogrel**	133201ACR, 133201ATB, 133201ATR, 133202ATB, 133203ATR, 506100ATB
**Cilostazol**	136901ATB, 492501ATB, 495201ATB, 498801ATB, 501501ATB
**Ticlopidine**	498900ATB, 239201ATB, 239202ATB
**Aspirin+Bethocarbamol**	256800ATB
**Aspirin+Clopidogrel**	517900ACH, 517900ACE, 517900ATE, 667500ACE
**Aspirin+Dipyridamole**	489700ACR

Table S2. ICD-10 codes of Charlson Comorbidity Index.

Comorbidities	Codes
**Myocardial infarction**	I21, I22, I252
**Congestive heart failure**	I43, I50, I099, I110, I130, I132, I255, I420, I425-I429, P290
**Peripheral vascular disease**	I70, I71, I731, I738, I739, I771, I790, I792, K551, K558, K559, Z958, Z959
**Cerebrovascular disease**	G45, G46, I60-69, H340
**Dementia**	F00-03, G30, F051, G311
**Chronic pulmonary disease**	J40-47, J60-67, I278-279, J701, J703, J684
**Rheumatologic disease**	M05-06, M32-34, M315, M351, M353, M360
**Peptic ulcer disease**	K25-28
**Mild liver disease**	B18, K73, 74, K700-703, K709, K713-715, K717, K760, K762-764, K768-769, Z944
**DM without complication**	E100-101, E106, E108-111, E116, E118-121, E126, E128-131, E136, E138-141, E146, E148-149
**DM with complication**	E102-105, E107, E112-115, E117, E122-125, E127, E132-135, E137, E142-145, E147
**Hemiplegia or paraplegia**	G81-82, G041, G114, G800, G830-834, G839
**Any malignancy**	C00-26, C30-C34, C37-41, C43, C45-58, C60-76, C81-88, C90-97
**Moderate to severe liver disease**	I850, I859, I864, I982, K704, K711, K721, K729, K765-767
**Metastatic tumor**	C77-80
**AIDS/HIV**	B20-22, B24.

Abbreviations: ICD-10, International Classification of Diseases, 10th revision, Clinical Modification; DM, diabetes mellitus; AIDS/HIV, acquired immune deficiency syndrome/human immunodeficiency virus.

Table S3. Cox regression analyses for patient survival according the use of RAS blockers and β-blockers in Groups 2 and 3.

	Univariate		Multivariate	
	**HR (95% CI)**	***P***	**HR (95% CI)**	***P***
Ref: NR-NB				
NR-B	0.97 (0.91–1.03)	0.336	1.06 (0.99–1.14)	0.112
R-NB	0.92 (0.87–0.97)	0.002	0.93 (0.87–0.99)	0.027
R-B	0.93 (0.88–0.98)	0.009	1.01 (0.95–1.07)	0.798
Ref: NR-B				
R-NB	0.94 (0.90–0.99)	0.033	0.88 (0.83–0.93)	<0.001
R-B	0.96 (0.91–1.01)	0.111	0.95 (0.90–1.01)	0.104
Ref: R-NB				
R-B	1.02 (0.97–1.06)	0.477	1.09 (1.03–1.14)	0.002

Multivariate analysis was adjusted for age, sex, underlying cause of ESRD, CCI score, type of vascular access, hemodialysis vintage, ultrafiltration volume, Kt/V_{urea}, hemoglobin, serum albumin, serum creatinine, serum phosphorus, serum calcium, systolic blood pressure, diastolic blood pressure, and use of aspirin and statin, and was performed using enter mode. **Abbreviations:** CCI, Charlson comorbidity index; CI, confidence interval; ESRD, end stage renal disease; HR, hazard ratio; RAS, renin-angiotensin system; NR-NB, patients without both two medications; NR-B, patients without RAS blockers and with β-blocker; R-NB, patients with RAS blockers and without β-blocker; R-B, patients with both two medications.

Table S4. Patients' clinical characteristics after weighting.

	**Group 1**	**Group 2**	**Group 3**	***P***
Age (years)	60.2 ± 0.1	60.5 ± 0.2	60.1 ± 0.1	0.124
Sex (male, %)	58.9%	58.7%	60.8%	0.004
Hemodialysis vintage (days)	1576 ± 12	1594 ± 19	1552 ± 16	0.396
Underlying cause of ESRD				0.029
Diabetes mellitus	43.9%	43.1%	45.1%	
Hypertension	25.9%	26.4%	25.8%	
Glomerulonephritis	10.4%	11.5%	10.6%	
Others	8.5%	8.3%	7.9%	
Unknown	11.2%	10.6%	10.5%	
CCI score	7.5 ± 0.0	7.6 ± 0.0	7.6 ± 0.0	<0.001
Follow-up duration (days)	1849 ± 6	1855 ± 10	1863 ± 8	0.592
Type of vascular access				0.309
Arteriovenous fistula	84.7%	84.4%	85.2%	
Arteriovenous graft	15.3%	15.6%	14.8%	
Kt/V_{urea}	1.53 ± 0.00	1.53 ± 0.00	1.53 ± 0.00	0.864
Ultrafiltration volume (L/session)	2.26 ± 0.01	2.28 ± 0.01	2.30 ± 0.01	0.055
Hemoglobin (g/dL)	10.7 ± 0.0	10.7 ± 0.0	10.6 ± 0.0	0.171
Serum albumin (g/dL)	3.99 ± 0.00	3.99 ± 0.00	0.99 ± 0.00	0.944
Serum phosphorus (mg/dL)	4.97 ± 0.01	4.98 ± 0.02	4.98 ± 0.01	0.644
Serum calcium (mg/dL)	8.9 ± 0.0	8.9 ± 0.0	8.9 ± 0.0	0.096
Systolic blood pressure (mmHg)	141 ± 0	141 ± 0	142 ± 0	0.011
Diastolic blood pressure (mmHg)	78 ± 0	78 ± 0	78 ± 0	0.809
Serum creatinine (mg/dL)	9.4 ± 0.1	9.5 ± 0.1	9.5 ± 0.1	0.235
Use of aspirin	42.3%	43.6%	44.1%	0.032
Use of stain	29.1%	30.4%	30.4%	0.078

Data are expressed as mean ± standard error for continuous variables and as percentages for categorical variables. **Abbreviations:** CCI, Charlson comorbidity index; ESRD, end-stage renal disease.

Table S5. Cox regression analyses for patient survival using weighted data

	Univariate		Multivariate	
	HR (95% CI)	*P*	HR (95% CI)	*P*
Group				
Ref: Group 1				
Group 2	1.08 (1.06–1.11)	<0.001	1.13 (1.08–1.18)	<0.001
Group 3	1.04 (1.02–1.06)	<0.001	1.07 (1.03–1.11)	<0.001
Ref: Group 3				
Group 2	1.04 (1.02–1.06)	<0.001	1.06 (1.01–1.10)	0.013

Multivariate analysis was adjusted for age, sex, underlying cause of ESRD, CCI score, type of vascular access, hemodialysis vintage, ultrafiltration volume, Kt/V_{urea}, hemoglobin, serum albumin, serum creatinine, serum phosphorus, serum calcium, systolic blood pressure, diastolic blood pressure, and use of aspirin and statin, and was performed using the enter mode. **Abbreviations:** CCI, Charlson comorbidity index; CI, confidence interval; ESRD, end stage renal disease; HR, hazard ratio.

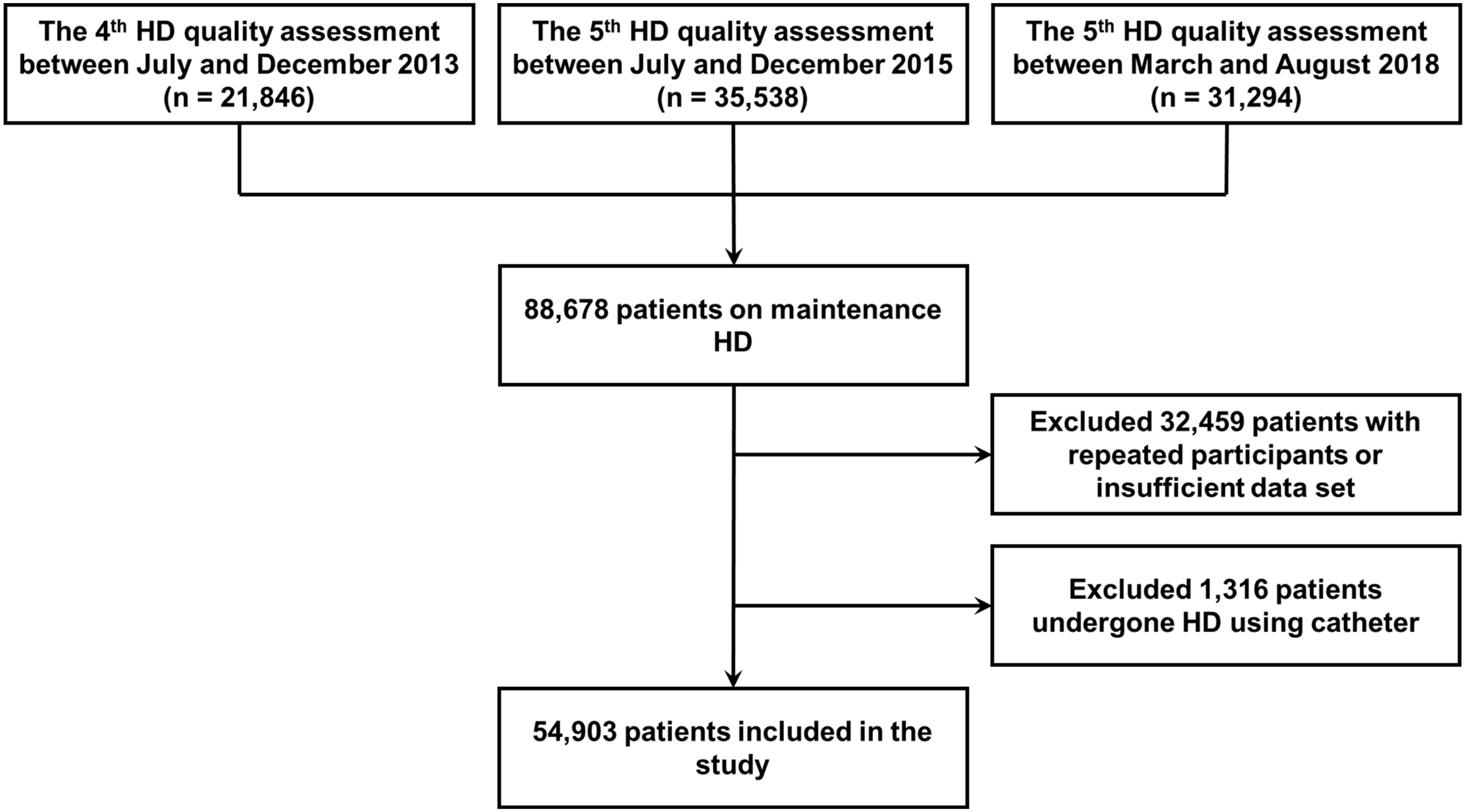

Figure S1. Study flow diagram.

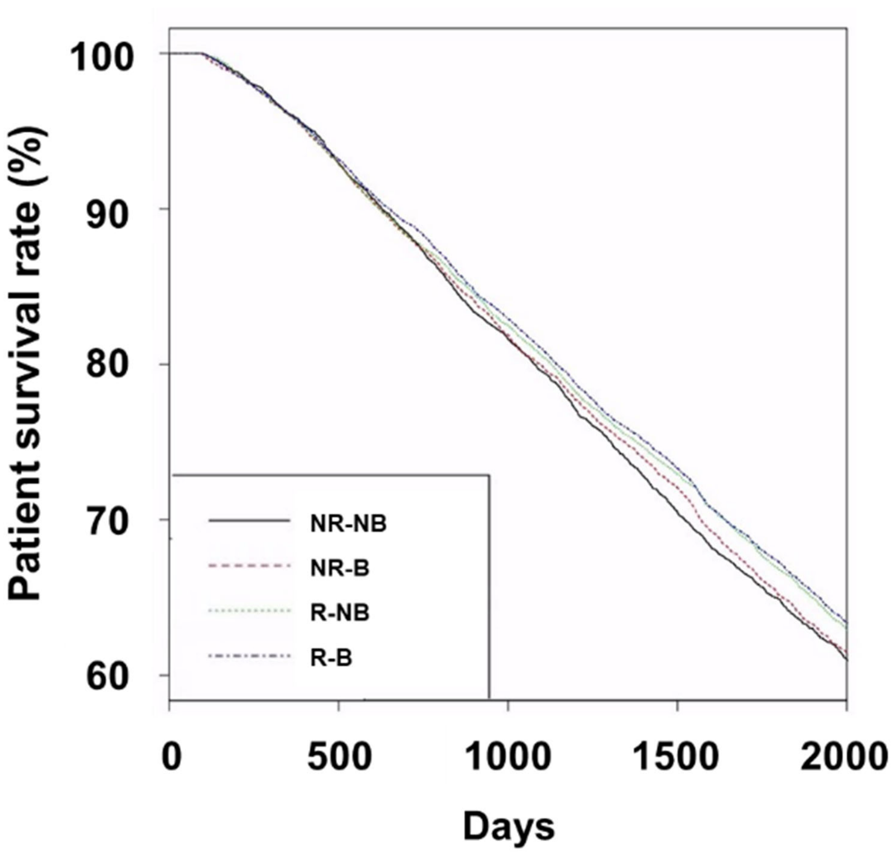

Figure S2. Kaplan–Meier curves of patient survival according the use of RAS blockers and β-blockers in Groups 2 and 3 ($P = 0.007$ for trend; $P = 0.406$ for NR-NB vs. NR-B; $P = 0.012$ for NR-NB vs. R-NB; $P = 0.025$ for NR-NB vs. R-B; $P = 0.059$ for NR-B vs. R-NB; $P = 0.172$ for NR-B vs. R-B; $P = 0.479$ for R-NB vs. R-B). **Abbreviations:** RAS, renin-angiotensin system; NR-NB, patients not on either of the two medications; NR-B, patients not on RAS blockers but were on β-blockers; R-NB, patients on RAS blockers but not on β-blockers; R-B, patients receiving both medications**.**

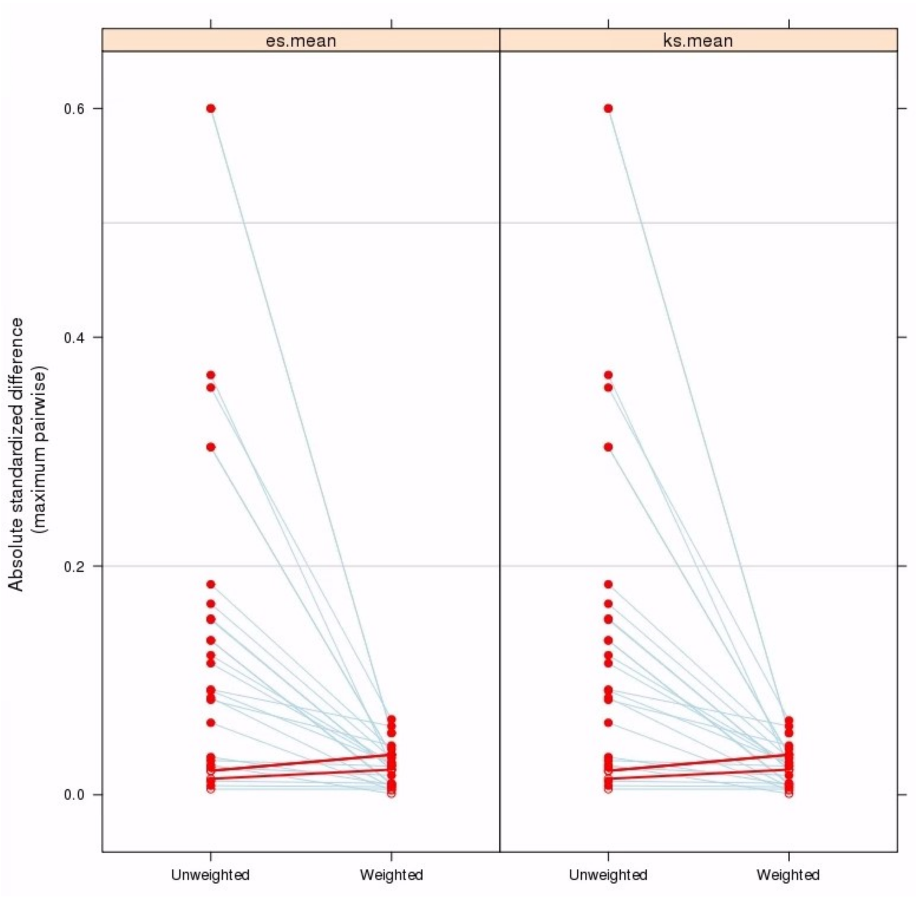

Figure S3. Absolute standardized difference plots for estimating propensity scores to generate weights. Left panel: effect size. Right panel: Kolmogorov–Smirnov statistics.

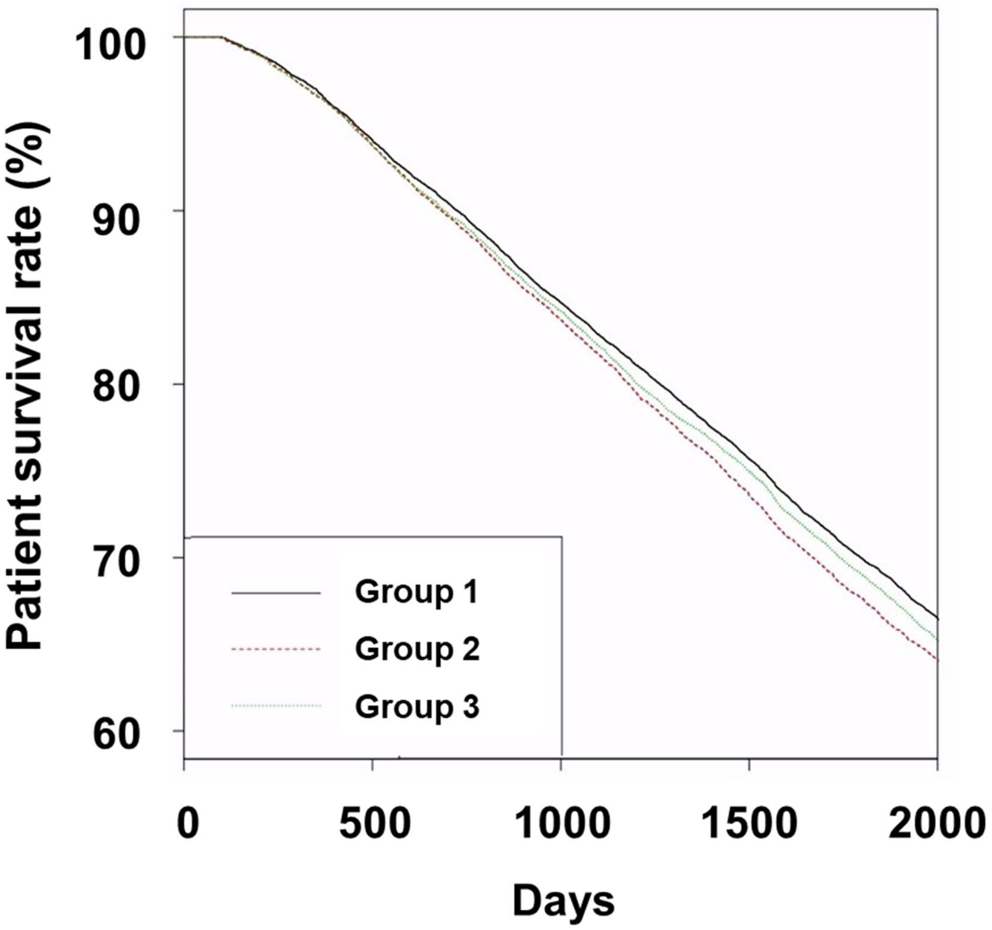

Figure S4. Kaplan–Meier curves of patient survival using weighted data according to the groups. The 5-year survival rates in Groups 1, 2, and 3 were 69.6%, 67.1%, and 68.6%, respectively ($P < 0.001$).
